# Workplace violence against frontline clinicians in emergency departments during the COVID-19 pandemic

**DOI:** 10.7717/peerj.12459

**Published:** 2021-11-23

**Authors:** Rui Liu, Yue Li, Ying An, Ling Zhang, Feng-Rong An, Jia Luo, Aiping Wang, Yan-Jie Zhao, Anzhe Yuan, Teris Cheung, Gabor S. Ungvari, Ming-Zhao Qin, Yu-Tao Xiang

**Affiliations:** 1 The National Clinical Research Center for Mental Disorders & Beijing Key Laboratory of Mental Disorders Beijing Anding Hospital & the Advanced Innovation Center for Human Brain Protection, Capital Medical University, Beijing, China; 2 Unit of Psychiatry, Department of Public Health and Medicinal Administration, & Institute of Translational Medicine, Faculty of Health Sciences, University of Macau, Macao SAR, China; 3 Department of Nursing, Beijing Tongren Hospital, Capital Medical University, Beijing, China; 4 Department of Emergency Medicine, Beijing Tongren Hospital, Capital Medical University, Beijing, China; 5 Eastside High School, Gainesville, FL, USA; 6 School of Nursing, Hong Kong Polytechnic University, Hongkong SAR, China; 7 Division of Psychiatry, School of Medicine, University of Western Australia, Perth, Australia; 8 University of Notre Dame Australia, Fremantle, Australia; 9 Department of Geriatric Medicine, Beijing Tongren Hospital, Capital Medical University, Beijing, China; 10 Centre for Cognitive and Brain Sciences, University of Macau, Macao SAR, China; 11 Institute of Advanced Studies in Humanities and Social Sciences, University of Macau, Macao SAR, China

**Keywords:** COVID-19, Emergency department, Workplace violence, China

## Abstract

**Background:**

Frontline clinicians working in emergency departments (ED) were at disportionate risk of workplace violence (WPV). We investigated the prevalence of WPV and its relationship with quality of life (QOL) in this group of health professionals in China during the COVID-19 pandemic.

**Methods:**

A cross-sectional, online study was conducted. The nine-item Workplace Violence Scale measured WPV.

**Results:**

A total of 1,103 ED clinicians participated in this study. The overall prevalence of WPV against ED clinicians was 29.2% (95% CI [26.5%-31.9%]). Having family/friends/colleagues infected with COVID-19 (Odds Ratio (OR) = 1.82, *P* = 0.01), current smoking (OR = 2.98, *P* < 0.01) and severity of anxiety symptoms (OR = 1.08, *P* < 0.01) were independently and positively associated with WPV, while working in emergency intensive care units (OR = 0.45, *P* < 0.01) was negatively associated with WPV. After controlling for covariates, clinicians experiencing WPV had a lower global QOL compared to those without (F_(1, 1103)_ = 10.9,*P* < 0.01).

**Conclusions:**

Prevalence of workplace violence against ED clinicians was common in China during the COVID-19 pandemic. Due to the negative impact of WPV on QOL and quality of care, timely preventive measures should be undertaken for ED clinicians.

## Introduction

Coronavirus disease 2019 (COVID-19) has become a major public health concern since early January, 2020 (World Health Organization, 2020a). By May 2020, over 5.9 million people have been infected with COVID-19 (World Health Organization, 2020b). To contain the rapid transmission of the novel virus, timely identification and treatment of COVID-19 is of crucial importantance (Chan et al., 2020; Shereen et al., 2020). Emergency departments (ED) play a critical role in early identification of infected cases (Bressan et al., 2020; National Health Commission of the People’s Republic of China , 2020), the provision of timely treatment and referral to other units/hospitals (Lam et al., 2016; Lam et al., 2019). Due to highly stressful and overcrowding work environment, heavy workload, limited communication between multidisciplinary team members, inadequate knowledge of the epidemic, lack of personal protective equipment and guidelines on the diagnosis and treatment for patients in the early stage of the COVID-19 pandemic, ED clinicians were exposed to an elevated risk of infection, burnout, mental health problems and even workplace violence (WPV) (Chapman & Styles, 2006; Gerberich et al., 2005; Ismail et al., 2020; Liu et al., 2020).

As a global public health challenge, WPV refers to physically and psychologically damaging actions against professionals in the workplace (National Institute for Occupational Safety and Health, 2016). Verbal and physical violence are common forms of WPV for ED clinicians (Jiao et al., 2015; Lu et al., 2020). A meta-analysis found that the lifetime prevalence of WPV against ED clinicians was 79.8% in China (Lu et al., 2020), the 1-year prevalence of WPV was 12.1% in their American counterparts (Speroni et al., 2014), and 89.9% in Beijing, China (Li et al., 2019) while the 2-year prevalence was 92.9% in Taiwan (Lee et al., 2020).The 1-month prevalence of verbal and physical WPV against WPV were 15.8% and 3.3%, respectively, while the corresponding figures for 3-month prevalence were 13.8% and 3.3% in China (Wang et al., 2019). WPV was found to be associated with diverse negative consequences, such as mental health problems, job dissatisfaction, decreased quality of patient care and medical errors (Gacki-Smith et al., 2009; Wu et al., 2014). In order to reduce the negative outcomes due to WPV, it is important to understand its patterns and correlates. In addition, no studies have examined the association between WPV and quality of life (QOL), a comprehensive health outcome, among ED clinicians during the COVID-19 pandemic. This gap in research has given the impetus to examine the prevalence of WPV and its associated factors in the frontline ED clinicians during the COVID-19 pandemic in China and explore the association between WPV and QOL.

## Methods

### Setting and participants

This cross-sectional study is part of a nationwide survey on mental health and related problems among clinicians during the COVID-19 pandemic conducted in all the 32 provinces, municipalities and autonomous regions of mainland China between March 15 and March 20, 2020 (Jin et al., 2021; Xie et al., 2021). Due to the risk of contagion, traditional face-to-face assessments and random sampling cannot be not adopted. Similar to other studies (An et al., 2020; Liu et al., 2020; Tian et al., 2020), data were collected using the WeChat-based QuestionnaireStar application (Changsha Renxing Science and Technology, Shanghai, China) using snowball sampling method. WeChat program is a widely used social communication platform with more than 1.2 billion users in China including all the ED clinicians we invited to participate in this study. To be eligible, participants were: (1) frontline ED clinicians during the study period; (2) aged ≥18 years; and (3) provide electronic informed consent. There were no exclusion criteria. The study was approved by the Medical Ethics Committee of Beijing Anding Hospital, China ((2020) KEYAN (No. 10) and (2020) KEYAN (No. 10) - 202024XG-1).

### Instruments

A data sheet was designed to collect demographic and clinical information including age, gender, education level, marital status, work experience, shift duty, rank (junior (*e.g.*, nursing asssistants, residents, senior medical officers) vs senior (*e.g.*, nursing manager, associate consultant and consultant)), type of hospital (non-tertiary (primary and secondary) vs tertiary), type of working unit, current smoking, and work experience (yes/no) during the 2003 SARS outbreak. Three additional COVID-19 related questions were also asked: (1) whether they provided direct care for COVID-19 patients; (2) whether they had family members, friends or colleagues infected with COVID-19; and (3) whether they lived in a province with more than 500 confirmed COVID-19 cases throughout the study period.

WPV experienced by frontline ED clinicians during the COVID-19 pandemic was measured by the workplace violence scale, Chinese version (Chen, 2011; Chen et al., 2004), which covers verbal and physical violence. This scale has satisfactory psychometric properties (Duan et al., 2019; Lu et al., 2019; Shi et al., 2020). Following previous studies (Lu et al., 2019; Xie et al., 2021), any verbal or physical violence was considered “experience of WPV” in this study.

Depressive symptoms were measured using the validated nine-item Patient Health Questionnaire (PHQ-9), Chinese version (Kroenke, Spitzer & Williams, 2001; Wang et al., 2014). The total score of the PHQ-9 ranges between 0 and 27, with a higher score indicating more severe depressive symptoms. Anxiety symptoms were assessed by the validated Chinese version of the Generalized Anxiety Disorder Scale-7 (GAD-7) (Spitzer et al., 2006). This scale has been widely used in Chinese populations (He et al., 2010; Tong et al., 2016). Its total score ranges between 0 and 21, with a higher total score indicating more severe anxiety. The sum of the first two items on the WHO Quality of Life Questionnaire-Brief Version (WHOQOL-BREF) (Harper, Power & Grp, 1998) assessed global QOL. A higher total score indicated better global QOL (Gholami et al., 2013).

### Statistics

Data analyses were performed with the SPSS statistical software, Version 24.0. Categorical variables were compared using Chi-square tests, and continuous variables were compared using *t*-tests, or Mann–Whitney *U* tests between ED clinicians with and without WPV. To explore factors independently associated with WPV, multiple logistic regression analysis was conducted with WPV as the dependent variable. All factors with significant group differences in the univariate analyses were entered as independent variables. Analysis of covariance (ANCOVA) was performed to compare global QOL between ED clinicians with and without WPV after adjusting for covariates. Level of significance was set as *P* < 0.05 and all tests were 2-tailed.

## Results

A total of 1,103 ED clinicians met the study entry criteria and participated in the study. The overall prevalence of WPV was 29.2% (95% Confidence interval (CI [26.5%–31.9%]; 322/1,103), with verbal violence of 27.5% (95%CI [24.8%–30.1%]; 303/1,103) and physical violence of 5.8% (95%CI [4.4%−7.2%]; 64/1,103) during the COVID-19 pandemic in China.

Table 1 presents the basic demographic information of the participants between the ED clinicians with and without violence (violence and non-violence groups, respectively). There were significant differences between the two groups in age, years of work experience, living circumstances, rank, work unit, shift duty, current smoking, having family/friends/colleagues infected with COVID-19, direct patient care of COVID-19 patients and PHQ-9 and GAD-7 total score (all *P* values <0.05). ANCOVA revealed that ED clinicians with WPV had a lower global QOL compared to those without (F_(1,1103)_ = 10.9, *P* < 0.01).

Table 2 shows the results of multiple logistic regression analysis. Having family/friends/colleagues infected with COVID-19 (Odds ratio (OR) = 1.82, 95%CI [1.13−2.92], *P* = 0.01), current smoking (OR = 2.98, 95%CI [1.57−5.67], *P* < 0.01) and more severe anxiety symptoms (OR = 1.08, 95%CI [1.02−1.14], *P* < 0.01) were positively associated with WPV. In contrast, working in emergency intensive care units (EICU) (OR = 0.45, 95%CI [0.33−0.62], *P* < 0.01) was negatively associated with WPV.

**Table 1:** Demographic characteristics of the study sample.

**Variables**	**Total (*N* = 1,103)**		**Non-violence group (*N* = 781)**		**Violence group (*N* = 322)**		**X** ^2^	**df**	***P***
	**N**	**%**	**N**	**%**	**N**	**%**			
Male gender	102	9.2	66	8.5	36	11.2	2.02	1	0.16
Married	710	64.4	495	63.4	215	66.8	1.14	1	0.29
College education and above	1073	97.3	756	96.8	317	98.4	2.34	1	0.13
Living with family	838	76.0	578	74.0	260	80.7	5.67	1	**0.02**
Junior (rank)	747	67.7	552	70.7	195	60.6	10.68	1	**<0.01**
Experience of 2003 SARS outbreak	184	16.7	130	16.6	54	16.8	0.003	1	0.96
Working in tertiary hospitals	961	87.1	681	87.2	280	87	0.01	1	0.91
Working in emergency intensive care	377	34.2	299	38.3	78	24.2	20.04	1	<0.01
Shift duty	929	84.2	670	85.8	259	80.4	4.92	1	**0.03**
Local COVID-19 cases ≥ 500	156	14.1	116	14.9	40	12.4	1.11	1	0.29
Having infected family/friends/colleagues	90	8.2	52	6.7	38	11.8	8.05	1	**<0.01**
Direct care of infected patients	250	22.7	157	20.1	93	28.9	10.03	1	**<0.01**
Current smoking	45	4.1	22	2.8	23	7.1	10.90	1	**<0.01**
	**Mean**	**SD**	**Mean**	**SD**	**Mean**	**SD**	**T/Z**	**df**	***P***
Age (years)	32.2	7.6	31.8	7.6	33.2	7.5	−2.77	1101	**<0.01**
Working experience (years)	10.7	8.3	10.3	8.3	11.8	8.3	−3.51^a^	–	**<0.01**
PHQ-9 total	4.9	5.4	4.0	4.8	7.1	6.0	−9.05^a^	–	**<0.01**
GAD-7 total	3.6	4.6	2.8	4.1	5.5	5.1	−9.41^a^	–	**<0.01**
Global QOL score	6.3	1.6	6.6	1.6	5.7	1.5	8.22	1101	**<0.01**

**Notes.**

a Mann–Whitney U test

Bolded values: *P* < 0.05.

SD standard deviation COVID-19 Corona Virus Disease 2019 SARS Severe Acute Respiratory Syndrome QOL Quality of Life GAD Generalized Anxiety Disorder PHQ-9 Patient Health Questionnaire

**Table 2:** Independent correlates of violence against ED clinicians (multiple logistic regression analysis).

**Variables**	**Multiple logistic regression analysis**		
	***P* value**	**OR**	**95% CI**
Living with family	0.27	1.23	0.85–1.78
Junior (rank)	0.06	0.69	0.47–1.01
Working in emergency intensive care	**<0.01**	0.45	0.33–0.62
Shift duty	0.28	0.79	0.51–1.22
Having infected family/friends/colleagues	**0.01**	1.82	1.13–2.92
Direct care of COVID-19 patients	0.06	1.36	0.99–1.88
Current smoking	**<0.01**	2.98	1.57 –5.67
Age (years)	0.45	0.97	0.90–1.05
Work experience (years)	0.60	1.02	0.95–1.09
GAD-7 total	**<0.01**	1.08	1.02–1.14
PHQ-9 total	**0.06**	1.05	1.00–1.10

**Notes.**

Bold values = *P*<0.05.

CI confidential interval GAD-7 Generalized Anxiety Disorder PHQ-9 Patient Health Questionnaire OR odds ratio

## Discussion

To the best of our knowledge, this was the first study that examined WPV among ED clinicians during the COVID-19 pandemic. The overall prevalence of WPV against ED clinicians was 29.2% (95% CI [26.5%-31.9%]) in this sutdy. Since no studies have used similar timeframe, direct comparison with findings of this study was not possible. For the sake of orientation, the 1-month prevalence of verbal and physical WPV against ED clinicians were 15.8% and 3.3%, respectively, while the corresponding figures for 3-month prevalence were 13.8% and 3.3% (Wang et al., 2019). Results of these existing studies were lower than the present findings, which were even higher than the 1-year prevalence of WPV against ED clinicians in the US (12.1%) (Speroni et al., 2014), but lower than the 2-year prevalence (92.9%) in Taiwan (Lee et al., 2020), and the 1-year prevalence (89.9%) in Beijing, China (Li et al., 2019). The frequency of WPV in this study was also lower than the lifetime prevalence (79.8%) in ED clinicians in China reported in a meta-analysis (Lu et al., 2020).

There are several reasons that could possibly explain the common WPV against ED clinicians during the COVID-19 pandemic. First, many ED clinicians, especially experienced physicians/nurses, joined the crisis response teams and volunteered to work in hospitals treating patients with COVID-19 infections, which exerted insurmountable pressure on existing scant health resources in China. In addition, low clinician-to-patient ratio, alongside with many cases suffering from life-threatening illnesses in ED that required immediate attention (Ajani, 2012; Chen et al., 2016), may have significantly affect the efficiency and quality of care, which is likely to have increased patients’ and their families’ dissatisfaction and irritability eventually erupting in WPV (Chen et al., 2016). Second, ED clinicians encountered enormous pressure and heavy workload during the pandemic. Excessive mental stress and physical exhaustion easily trigger mental health problems (Li et al., 2020; Xiang et al., 2020), which, together with use of personal protective equipment hindered effective communication with patients, or stirred up conflicts with patients and/or their family members (Li et al., 2019). Third, urgent contingent measures in ED were adopted to prevent the rapid transmission of COVID-19. For example, all patients and their families had to wear facemasks with temperature check on entry and the entrance and exist doors to ED were limited, which increased disputes between hospital administrators, physicians/nurses and patients, together with long waiting times and high medical expenses. All these human and structural factors contributed to the high frenquency of WPV (Liu et al., 2015; Wu et al., 2012).

ED clinicians working in EICU were less likely to report WPV in this study. In EICU, ED clinicians had sufficient time to communicate with patients about their families and adjust treatment plans (Briones, 2016; Michel & Walston, 2018). Furthermore, most family visits were suspended during the COVID-19 pandemic. This reduced the likelihood of face-to-face WPV originated from patients’ families (Sharifi et al., 2020). Besides, emergency psychological response services established for EICU in many hospitals could help alleviate patients’ psychological distress and other mental health problems (Ahmad et al., 2020; Kang et al., 2020; Li et al., 2020; Xiang et al., 2020; Yang et al., 2020), which further reduced the risk of WPV in ED settings.

In this study, ED clinicians who had family/friends/colleagues infected with COVID-19 reported more WPV than those without. Frontline clinicians with infected family/friends/colleagues experienced more fear of contagion and other negative mood symptoms, such as high level of stress, depressive and anxiety symptoms and psychological trauma (Kumar & Nayar, 2020). Psychological trauma was common among healthcare workers with infected family/friends/colleagues during the Severe Acute Respiratory Syndrome (SARS) outbreak (Wu et al., 2009). Clinicians’s negative attitude could affect the overall quality of service delivery and impair effective communication with patients and their families, a contributing factor WPV.

Clinicians who smoked reported more WPV than non-smokers (Arnetz, Arnetz & Petterson, 1996; Borrello, 2012), which was also confirmed in this study. Smoking is associated with high level of work-related stress (Roberts & Grubb, 2014) and burnout (Koutsimani, Montgomery & Georganta, 2019; Roberts & Grubb, 2014), which negatively affects concentration, attention to patients and then increases the risk of medical errors, resulting in poor relationship with patients and high risk of WPV. Similar to previous findings (Cheung & Yip, 2017; Jiao et al., 2015), ED clinicians suffering from severe anxiety symptoms were at higher risk of WPV. The relationship between anxiety and WPV is bidirectional. Anxious ED clinicians are more likely to stir up conflicts with others, resulting in aggression and WPV (Cheung & Yip, 2017). Clinicians’ anxiety affects the quality of care, which could trigger WPV perpetrated by patients and/or their family members (Chen et al., 2008; Pourshaikhian et al., 2016).

Health professionals suffer from short- and long-term adverse consequences following WPV incidents such as, physical injuries, and emotional problems leading to poor quality of care (Magnavita, 2014; Mento et al., 2020). Therefore, it is reasonable to assume that ED clinicians who experienced WPV were more likely to have lower QOL than those without as was found that in this study echoing previous findings (Lu et al., 2020; Nowrouzi-Kia, 2017; Wu et al., 2014).

The merits of this study included the large sample size and use of standardized instruments on WPV. However, there were several limitations that needed to be addressed. First, being a cross-sectional study, the causal relationships between variables could not be established. Second, for logistical reasons, factors potentially related to workplace violence (*e.g.*, clinician-patient relationship, social support, and participants’ preexisting psychiatric, or psychological and/or medical conditions) were not recorded. Third, most of ED clinicians were females, which constitutes gender bias distorting the results to an unknown extent. Finally, Macau, Hong Kong and Taiwan were not included in the study due to their different health service systems from those of mainland China. Finally, the sample size in each province, municipality and autonomous region was not recorded, which would also bias the results.

## Conclusions

WPV against ED clinicians was common during the COVID-19 pandemic in China. Due to the detrimental impact of WPV on patient care and clinicians’ QOL, effective preventive measures targeting WPV should be developed and timely psychological assistance should be provided to victims of WPV. Special social support and psychological crisis interventions should be offered to ED clinicians who have family/friends/colleagues infected with COVID-19 (Lai et al., 2020). In addition, health authorites should develop stragies to lower the risk of WPV by creating safe working environment, to increase clinican-patient ratio, to reduce the working hours of health workers, and to set up education and training program on prevention of WPV (Ghareeb, El-Shafei & Eladl, 2021; Liu et al., 2019). Furthermore, as the negative effects of WPV on clinicains’ physical and psychological health and job satisfaction may persist, regular follow-up assessments on their stress level and mental health should be conducted (Byon et al., 2021; Gu et al., 2021; Pan et al., 2020).

## Supplemental Information

10.7717/peerj.12459/supp-1 Supplemental Information 1 Raw data

10.7717/peerj.12459/supp-2 Supplemental Information 2 Updated questionnaires

## Additional Information and Declarations

### Data Availability

The following information was supplied regarding data availability: The raw measurements are available in the Supplementary File.
