# Supplementary material for: Workplace violence against frontline clinicians in emergency departments during the COVID-19 pandemic
Source: PeerJ. 2021 Nov 23;9:e12459. doi: 10.7717/peerj.12459 (PMC8621782; doi:10.7717/peerj.12459)
Supplement: Supplemental Information 2 [file peerj-09-12459-s002.docx]

尊敬的精神科、急诊科、眼科和耳鼻喉科护理同道们:

您好,本调查旨在了解抗击新型冠状病毒疫情中相关临床学科和科室护理工作者的精神心理健康状况，为今后开展相关职业健康教育、服务和护理培训提供依据。请您按照您的情况如实填写，问卷均以匿名形式提交，我们将严格保护您的信息，不会外泄您的个人资料。十分感谢您支持和参与！谢谢！

**1. GAD-7焦虑症筛查量表**

**请根据您最近一周的情况进行回答，如果一周内曾有波动，请以目前情况为准：**

|  | **完全不会** | **好几天** | **一半以上的天数** | **几乎**  **每天** |
| --- | --- | --- | --- | --- |
| 1. 感觉紧张，焦虑或急切 | 0 | 1 | 2 | 3 |
| 2. 不能够停止或控制担忧 | 0 | 1 | 2 | 3 |
| 3. 对各种各样的事情担忧过多 | 0 | 1 | 2 | 3 |
| 4. 很难放松下来 | 0 | 1 | 2 | 3 |
| 5. 由于不安而无法静坐 | 0 | 1 | 2 | 3 |
| 6. 变得容易烦恼或急躁 | 0 | 1 | 2 | 3 |
| 7. 感到似乎将有可怕的事情发生而害怕 | 0 | 1 | 2 | 3 |

**2.生活质量**

1、您怎样评估您的生活质量？

1= 极不满意; 2=不满意; 3=没有满意或不满意; 4=很满意; 5=极满意

2、您满意自己目前的健康状况吗？

1= 极不满意; 2=不满意; 3=没有满意或不满意; 4=很满意; 5=极满意

**3.** 本次疫情爆发以来**，**您有无经历过：

| **语言攻击:** | | | | | |
| --- | --- | --- | --- | --- | --- |
| Verbal 1) | 责骂、谩骂、辱骂、贬低或其它有损个人尊严的言语(当面、书信或传单等)，但没有身体接触？ | 0＝ 无； | 1＝ 1次； | 2= 2-3次； | 3＝ >3次 |
| Verbal 2) | 威胁(言语、吐口水、挥拳、拍桌、跺脚、拿东西对着你,也包括电话、文字、第三人传话，扬言投诉等), 但没有身体接触？ | 0＝ 无； | 1＝ 1次； | 2= 2-3次； | 3＝ >3次 |
| **躯体攻击:** （以身体接触或用东西攻击），包括打、踢、拍、扎、推、咬、掷物、拧胳膊、拽头发等 | | | | | |
| Physical 1) | 躯体攻击,没有造成躯体损伤 | 0＝ 无； | 1＝ 1次； | 2= 2-3次； | 3＝ >3次 |
| Physical 2) | 造成轻度损伤,如疼痛、擦伤、刮伤或疲瘫 | 0＝ 无； | 1＝ 1次； | 2= 2-3次； | 3＝ >3次 |
| Physical 3) | 造成明显损伤,如伤口、骨折、内脏或头部损伤 | 0＝ 无； | 1＝ 1次； | 2= 2-3次； | 3＝ >3次 |
| Physical 4) | 造成严重后果,如功能障碍或永久性残疾 | 0＝ 无； | 1＝ 1次； | 2= 2-3次； | 3＝ >3次 |

**4. PHQ-9抑郁症筛查量表**

**请根据您最近一周的情况进行回答，如果一周内曾有波动，请以目前情况为准：**

|  | **完全不会** | **好几天** | **一半以上的天数** | **几乎每天** |
| --- | --- | --- | --- | --- |
| 1.做事时提不起劲或没有兴趣 | 0 | 1 | 2 | 3 |
| 2.感到心情低落、沮丧或绝望 | 0 | 1 | 2 | 3 |
| 3.入睡困难、睡不安稳或睡眠过多 | 0 | 1 | 2 | 3 |
| 4.感觉疲倦或没有活力 | 0 | 1 | 2 | 3 |
| 5.食欲不振或吃太多 | 0 | 1 | 2 | 3 |
| 6.觉得自己很糟，或觉得自己很失败。或让自己或家人失望 | 0 | 1 | 2 | 3 |
| 7.对事物专注有困难，如读报纸或看电视时不能集中注意力 | 0 | 1 | 2 | 3 |
| 8.动作或说话速度缓慢到别人已经觉察？或正好相反，烦躁或坐立不安、动来动去的情况更胜于平常 | 0 | 1 | 2 | 3 |
| 9.有不如死掉或用某种方式伤害自己的念头 | 0 | 1 | 2 | 3 |

Dear psychiatric, emergency, ophthalmology and ENT care colleagues：

Hello, this survey aims to understand the relevant clinical disciplines in the fight against the new coronavirus epidemic. The mental and psychological health status of nursing staff in the department and the department provides a basis for the development of relevant occupational health education, services and nursing training in the future. Please fill in the questionnaires truthfully according to your situation. The questionnaires are submitted anonymously. We will strictly protect your information and will not leak your personal information. Thank you very much for your support and participation! Thank you!

1. GAD-7

Over the last one weeks, how often have you been bothered by any of the following problems?

|  | Not at all | Several days | More than half the days | Nearly every day |
| --- | --- | --- | --- | --- |
| 1. Feeling nervous, anxious or on edge? | 0 | 1 | 2 | 3 |
| 1. Not being able to stop or control worrying? | 0 | 1 | 2 | 3 |
| 1. Worrying too much about different things? | 0 | 1 | 2 | 3 |
| 1. Trouble relaxing? | 0 | 1 | 2 | 3 |
| 1. Being so restless that it is hard to sit still? | 0 | 1 | 2 | 3 |
| 1. Becoming easily annoyed or irritable? | 0 | 1 | 2 | 3 |
| 1. Feeling afraid as if something awful might happen? | 0 | 1 | 2 | 3 |

1. Quality of life

|  | Very poor | Poor | Neither poor nor good | Good | Very good |
| --- | --- | --- | --- | --- | --- |
| 1.How would you rate your quality of life? | 1 | 2 | 3 | 4 | 5 |
| 2.How satisfied are you with your health? | 1 | 2 | 3 | 4 | 5 |

1. Workplace violence scale

During the COVID-19 outbreak, have you ever experienced:

|  |  | None | Once | 2-3 times | >3 times |
| --- | --- | --- | --- | --- | --- |
| Verbal | | | | | |
| Verbal 1） | Verbal abuse, humiliates, degrades or otherwise indicates a lack of respect for the dignity and worth of an individual, but no physical contact. | 0 | 1 | 2 | 3 |
| Verbal 2） | Threats (verbal, spit, punching, slapping, stomping, point somebody with something, including phone, text, third party messenger, threatened complaints), but no physical contact? | 0 | 1 | 2 | 3 |
| Physical assault  Includes beating, kicking, slapping, stabbing, shooting, pushing, biting, pinching, among others. | | | | | |
| Physical 1) | Physical assault without body injury | 0 | 1 | 2 | 3 |
| Physical 2) | Physical assault causing mild injuries, such as pain, scratches, swelling, and bruises | 0 | 1 | 2 | 3 |
| Physical 3) | Physical assault causing apparent injuries, such as wounds, fracture, and visceral or brain injury | 0 | 1 | 2 | 3 |
| Physical 4) | Physical assault causing severe injuries, such as functional disorder or disability. | 0 | 1 | 2 | 3 |

4. PHQ-9

Over the past 2 weeks, how often have you been bothered by any of the following problems?

|  | Not at all | Several days | More than half the days | Nearly every day |
| --- | --- | --- | --- | --- |
| 1.Little interest or pleasure in doing things. | 0 | 1 | 2 | 3 |
| 2.Feeling down, depressed or hopeless. | 0 | 1 | 2 | 3 |
| 3.Trouble falling asleep, staying asleep, or sleeping too much. | 0 | 1 | 2 | 3 |
| 4.Feeling tired or having little energy. | 0 | 1 | 2 | 3 |
| 5.Poor appetite or overeating. | 0 | 1 | 2 | 3 |
| 6.Feeling bad about yourself - or that you’re a failure or have let yourself or your family down. | 0 | 1 | 2 | 3 |
| 7.Trouble concentrating on things, such as reading the newspaper or watching television. | 0 | 1 | 2 | 3 |
| 8.Moving or speaking so slowly that other people could have noticed. Or, the opposite - being so fidgety or restless that you have been moving around a lot more than usual. | 0 | 1 | 2 | 3 |
| 9.Thoughts that you would be better off dead or of hurting yourself in some way. | 0 | 1 | 2 | 3 |
